# Effect of YH0618 soup on chemotherapy-induced toxicity in patients with cancer who have completed chemotherapy: study protocol for a randomized controlled trial

**DOI:** 10.1186/s13063-016-1443-9

**Published:** 2016-07-26

**Authors:** Jie-shu You, Jian-ping Chen, Jessie S.M. Chan, Ho-fun Lee, Mei-kuen Wong, Wing-Fai Yeung, Li-xing Lao

**Affiliations:** 1 School of Chinese Medicine, The University of Hong Kong, 10 Sassoon Road, Pokfulam, Hong Kong; 2 Department of Social Work & Social Administration, The University of Hong Kong, SAR, Hong Kong; 3 Department of Clinical Oncology, The University of Hong Kong, SAR, Hong Kong

**Keywords:** YH0618, Chemotherapy-induced toxicity, Taxanes, Anthracyclines, Dermatologic toxicity, Fatigue, Study protocol

## Abstract

**Background:**

The incidence of cancer has been staying at a high level worldwide in recent years. With advances in cancer diagnosis and therapy strategy, the survival rate of patients with cancer has been increasing, but the side effects of these treatments, especially chemotherapy, are obvious even when the chemotherapy ceases. YH0618, a prescription, has showed efficacy in reducing chemotherapy-induced toxicity through long clinical practice. However, there is no scientific research exploring the effects of YH0618 in patients with cancer. Therefore, using a randomized controlled trial, this study will explore the efficacy of YH0618 on ameliorating chemotherapy-induced toxicity including dermatologic toxicity, myelosuppression, hepatotoxicity and nephrotoxicity and improving fatigue in cancer patients who have completed chemotherapy.

**Methods/design:**

This is a prospective assessor-blinded, parallel, randomized controlled trial. Patients with cancer at any stage who have completed chemotherapy within two weeks will be randomly divided into group A (YH0618) and group B (wait-list) using a 1:1 allocation ratio. The chemotherapeutic agents include taxanes or anthracyclines. Subjects assigned to group A will receive YH0618 soup 6 days a week for 6 weeks and uncontrolled follow-up for 6 weeks, while group B are required to wait for 6 weeks before receiving YH0618 intervention. The primary outcome of this study is the incidence of protocol-specified grade ≥2 dermatologic toxicities graded by NCI CTCAE Chinese version 4.0 and changes of fingernail color, face skin color and tongue color evaluated by the L*a*b system within 6 weeks. There are some secondary outcomes associated with dermatologic toxicity including fatigue and clinical objective examination.

**Discussion:**

There are few scientific and safe methods in ameliorating chemotherapy-induced toxicity. The proposed study may provide direct and convincing evidence to support YH0618 as an adjuvant treatment for reducing chemotherapy-induced toxicity, which could be introduced into clinical settings.

**Trial registration:**

Chinese Clinical Trial Registry: ChiCTR-IOR-15006486. Registered on 21 May 2015.

**Electronic supplementary material:**

The online version of this article (doi:10.1186/s13063-016-1443-9) contains supplementary material, which is available to authorized users.

## Background

According to the data from the WHO’s World Cancer Report 2014, there are an estimated nearly 25 million cancer cases that will occur over the next two decades worldwide, a rise of 75 % [1]. Because of advances in cancer diagnosis and treatment, the survival rate of patients with cancer has been increasing, but the side effects of these treatments, especially chemotherapy, are obvious [2]. Chemotherapy, as a standard regimen, has been used for treating cancers for 70 years. However, in recent years, there is a controversy as to whether it should continue to be used because it causes major side effects, especially toxicity. However, as there is no better regimen to replace chemotherapy, searching for safe and effective ways to ameliorate chemotherapy-induced toxicity has become an urgent issue in the cancer research area.

Chemotherapy-induced dermatologic toxicity is one of the most obvious toxicities, and it can cause considerable cosmetic concern, pain, infection, and functional impairment, and it further impact patients’ quality of life, although it is rarely life-threatening [3, 4]. Its symptoms mainly include pruritus, bullous dermatitis, dry skin, skin pain, paronychia, skin infection, acneiform rash, alopecia, nail discoloration, and nail loss [5]. Low-grade symptoms, such as nail ridging and nail discoloration, can impact beauty and quality of life. Medium-grade symptoms like painful blisters and intense pruritus may also interfere with treatment, resulting in dose reduction or interrupting treatments. Some symptoms may even continue after treatment ceases. Dermatologic toxicity is a major side effect of more than 30 anticancer agents, such as antimetabolites, hormonal agents, topoisomerase-interacting agents, and taxanes; of these taxanes and anthracyclines are the main chemotherapy drugs causing these symptoms [6, 7]. Taxanes are microtubule-stabilizing agents, including paclitaxel, docetaxel, and cabazitaxel, which can stabilize GDP-bound tubulin in the microtubule, therefore interrupting cell division and causing cell death [8]. Anthracyclines are one of the most effective chemotherapeutic drugs and are used to treat many cancers, like lymphomas, leukemias, breast cancer, uterine cancer, ovarian cancer, and lung cancer. The mechanisms of anthracyclines include four pathways. Anthracyclines directly inhibit the synthesis of DNA and RNA by inserting themselves in between nucleotides, thereby interfering with replication and transcription [9]. They also inhibit topoisomerase II, thereby generating reactive oxygen species that damage DNA and cell membranes, or preventing the relaxing of supercoiled DNA and thus blocking DNA transcription and replication [10]. They can also induct histone eviction from chromatin, deregulating DNA damage response, epigenomes, and transcriptomes [11].

Recently, multiple options for reducing chemotherapy-induced dermatologic toxicity have been investigated, but there are no acknowledged and standard treatments. Lacouture et al. [4] found the incidence of grade 2 skin toxicities during the 6-week skin treatment period was 29 % in the pre-emptive group and 62 % in the reactive group. A prospective study demonstrated that ice packs could reduce the incidence of hand-foot syndrome in patients treated with liposomal doxorubicin [5]. In contrast, no significant differences were determined between hand conditions in terms of time to event, nor in terms of toxicity, in cryotherapy and control groups in a randomized control trial [12]. Oral pyridoxine also failed to prevent capecitabine-associated hand-foot syndrome [13].

Chemotherapy-induced myelosuppression, hepatotoxicity, and nephrotoxicity are also common, and they are significant complications affecting the treatment of cancer patients. Moreover, from the view of Chinese medicine, the liver and kidneys are the main organs responsible for detoxification and purification of the blood. Deficiencies of the liver and kidneys lead directly to fatigue, which is also an obvious symptom of cancer patients who just completing chemotherapy. So we believe that dermatologic toxicity and fatigue are a reflection of hepatotoxicity and nephrotoxicity.

Recent attempts to reduce toxicity by using individual compounds have been unsatisfactory [14], and there is also concern about whether using Chinese herbs will cause hepatotoxicity and nephrotoxicity or interactions with anticancer agents [15]; therefore, medical food is the best choice. YH0618, a prescription, has showed efficacy in reducing chemotherapy-induced toxicity through long clinical practice. YH0618 consists of four medical foods (such as black soybeans) which are recommended by clinicians for patients with cancer, and all components have a history of safe use in other foods. Besides, each of the components possesses a distinct pharmacological profile, including removing free radicals in the body, regulating the immune system, preventing cancer, detoxifying, and enhancing taste, etc. [16–18]. Black soybean, as the monarch drug in the prescription, has been used for detoxification over the millennia in China. Modern research has further explored its active compounds and mechanisms in detoxification. Liao et al. [19] found that a novel polysaccharide of black soybean could improve 5-flurouracil- and irradiation-induced myelosuppression in animal experiments. Anthocyanins, which are the main active compounds in the black soybean seed coat, are water-soluble and possess significant anticancer [20], antioxidation [21], liver-protective [22, 23], and kidney-protective effects [24] in vitro and in vivo experiments. Over long clinical observation, YH0618 soup has been proved to be effective in reducing chemotherapy-induced toxicity by clinical observation, but there is not yet any scientific research exploring the effects of YH0618 soup in patients with cancer. Therefore, this study will be conducted to further verify the benefits of YH0618: the hypothesis is that YH0618 soup would ameliorate chemotherapy-induced toxicity including dermatologic toxicity, hematotoxicity, hepatotoxicity, and nephrotoxicity in cancer patients who have completed chemotherapy. Moreover, since little research has explored the role of medical food intervention in reducing chemotherapy-induced toxicity, and YH0618 is a safe medical food, the project will bring huge benefits to patients with cancer. The YH0618 prescription has the potential to become a standard alternative intervention strategy in providing care to these patients.

## Methods/design

### Study design

**Fig. 1:**
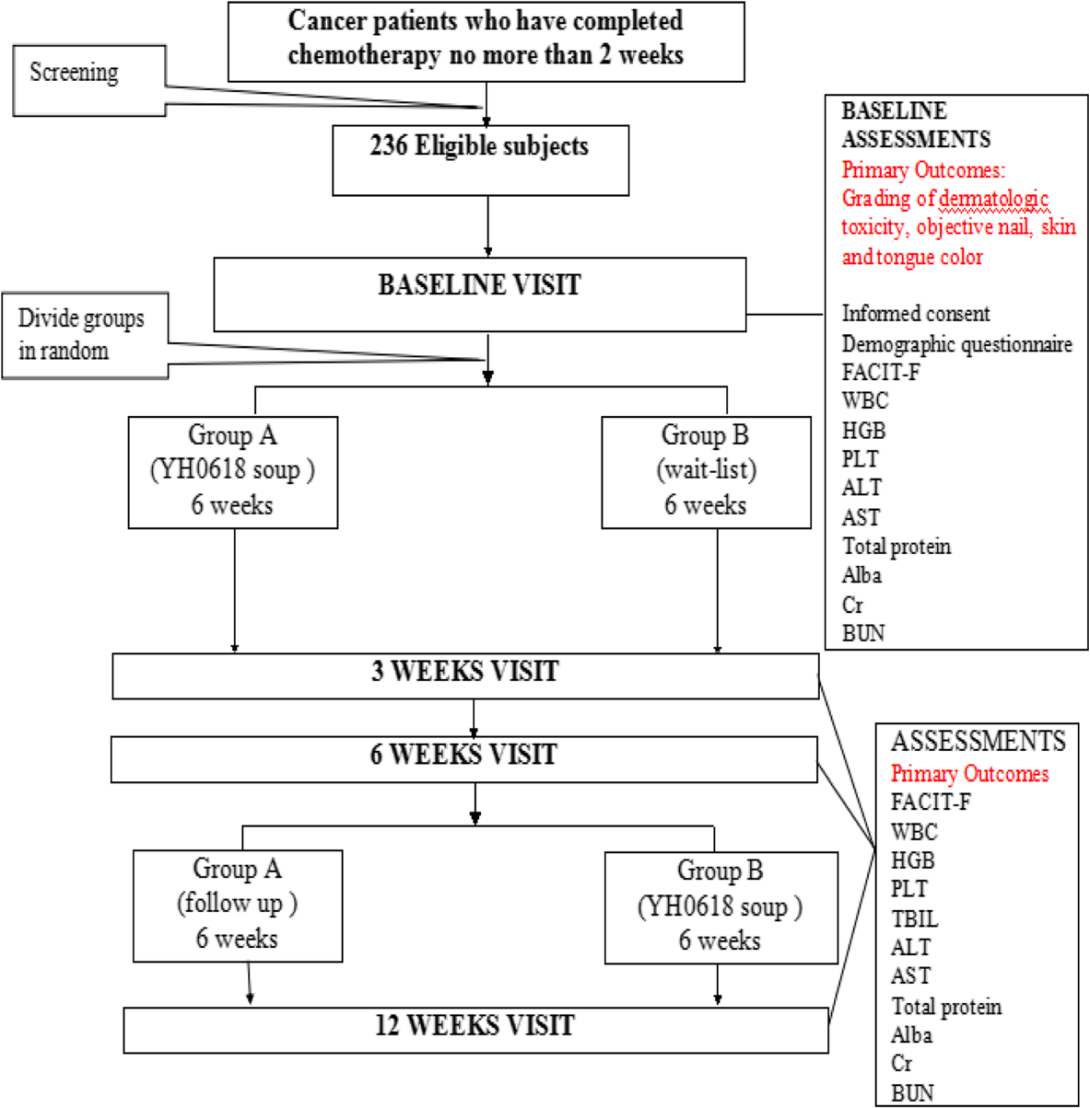
Flow chart of the clinical trial

This is a randomized controlled trial which aims at determining whether YH0618 would be more effective than the wait-list control conditions in ameliorating chemotherapy-induced dermatologic toxicity, blood system disorders, and hepatic and renal disorders and in improving fatigue in cancer patients who have completed chemotherapy. To achieve this goal, a total of 236 patients with cancer at any stage who have completed chemotherapy within 2 weeks will be recruited for the study. The patients will be randomly divided into group A (YH0618) and group B (wait-list) using a 1:1 allocation ratio, adhering to the Consolidated Standards of Reporting Trials (CONSORT) statement [25] and the Standard Protocol Items: Recommendations for Interventional Trials (SPIRIT) statement [26]. The measures of clinical outcomes mainly include the grading of dermatologic toxicity, skin color, nail color, tongue color, and score of fatigue. Several biomarkers associated with myelosuppression, hepatotoxicity, and nephrotoxicity in the blood will also be measured. The flow chart of the study is shown in Fig. 1, and the trial process is listed in Table 1. The evaluation and data collected will be conducted at the clinics of the School of Chinese Medicine, HKU.

**Table 1:** Trial process chart

	Before baseline screening	Baseline	Visit 1 Treatment phase	Visit 2 Treatment phase	Visit 3 Follow-up phase
Process items		0 day	3 weeks	6 weeks	12 weeks
Patients					
Inclusion and exclusion criteria	×				
Informed consent		×			
Demographics		×			
Medical history	×	×			
Randomization and allocation concealment		×			
Primary outcomes					
Grading of dermatologic toxicity		×	×	×	×
Objective skin, nail, and tongue color (L*a*b)		×	×	×	×
Secondary outcomes					
Fatigue measurement score		×	×	×	×
Routine blood test (WBC, HGB, PLT)		×	×	×	×
Liver function (ALT, AST, total protein, Alba)		×	×	×	×
Renal function (Cr and BUN)		×	×	×	×
Adverse events			×	×	×
Patients’ diary records		Every day during the study			

### Ethics

Ethical approval of the study protocol was obtained from the Institutional Review Board of the University of Hong Kong/Hospital Authority Hong Kong West Cluster (HKU/HA HKW IRB, reference no. UW15-023). The trial was registered in the Chinese Clinical Trials Registry: ChiCTR-IOR-15006486. Patients will receive a detailed information sheet and complete written consent forms.

The trial is managed by the School of Chinese Medicine, HKU, and data will be supervised by the Data and Safety Monitoring Board (DSMB). Study documents (digital and hard copies) will be retained in a secure location for 5 years after trial completion.

Under the laws of Hong Kong, patients enjoy or may enjoy rights for the protection of the confidentiality of their personal data, such as those regarding the collection, custody, retention, management, control, use (including analysis or comparison), transfer in or out of Hong Kong, non-disclosure, erasure and/or any way of dealing with or disposing of any of their personal data in or for this study. Clinical trial insurance will be provided to the subjects.

### Subjects

A total of 236 eligible patients will be recruited through advertisements. Inclusion criteria include: (1) patients with cancer of any stage aged between 18–75; (2) completed chemotherapy no more than 2 weeks ago; (3) received chemotherapeutic agents containing taxanes or anthracyclines; and (4) a life expectancy of at least 6 months. Exclusion criteria are: (1) subjects with a medical history of dermatosis; (2) any treatment for detoxification within 3 months; (3) any severe mental disorders or history of psychiatric illness or taking psychotropic drugs; (4) severe lesions of liver or kidney; (5) pregnancy or potential pregnancy; (6) allergies to some specific foods, like black soybean, etc. Eligible patients will be invited to participate in this study after providing their written consent form. All participants will be closely monitored in the study. These patients are given open clinical treatment as an additional safeguard.

### Estimation of sample size

The primary outcome in this study is the proportion of subjects with chemotherapy-induced dermatologic toxicity as measured by National Cancer Institute Common Terminology Criteria for Adverse Events (NCI CTCAE) specific grade ≥2. According to previous research, the incidence of NCI CTCAE specific grade ≥2 after the intervention is about 30 % [12]. Our pilot study showed that about 10 % of subjects would have NCI CTCAE specific grade ≥2 after taking the YH0618 soup; thus, the difference in proportion of subjects with dermatologic toxicity between the soup group and waiting-list control group is 20 %. The difference in proportion between the two groups will be examined by a Z-test. To achieve a type I error alpha = 0.05 and power (1 – beta) = 90 %, the minimal number of subjects needed in each group is 82. We estimated a 30 % attrition rate at the end of follow-up; hence, a sample size of at least 118 in each group (236 in total) is planned for this study.

### Randomization and blinding

The research assistant will give each patient a unique study number immediately after they complete the written consent. A computer random digit (randomization list) will be generated by a statistician not involved in this study. Codes representing the two groups will be printed on one A4 opaque paper, and then be put into a envelope which is blotted out using double sided tape. The tape should be torn by the patients themselves just after completing the baseline testing. Because this trial compares the effects of the YH0618 group and the wait-list group, it is not possible to blind the patients; thus, they know which group they are in. However, the randomization sequence and different groups will be kept hidden from the practitioners, data collectors, and statisticians.

### Intervention and control conditions

Prior to intervention, baseline data will be collected including demographics, chemotherapy-induced dermatologic toxicity, skin color, nail color, tongue color, score of fatigue, and the indexes of myelosuppression, hepatotoxicity, and nephrotoxicity in the blood. After that, participants assigned to group A will receive YH0618 soup 6 days/week for 6 weeks and uncontrolled follow-up for 6 weeks, while group B are required to wait for 6 weeks and then receive YH0618 soup for 6 weeks. There is no contraindication between YH0618 soup and chemotherapy, because all the components are general food which people often eat in their daily lives.

### Outcome evaluation

#### Primary outcome

The primary outcome of this study is the incidence of specific grade ≥2 dermatologic toxicities and changes of nail color, skin color, and tongue color after 6 weeks of intervention which will be graded by NCI CTCAE Chinese version 4.0 and evaluated by the L*a*b system, respectively.

##### Grading of dermatologic toxicity

NCI CTCAE is commonly used to monitor and rate the severity of chemotherapy-induced toxicity [27]. The CTCAE displays grades 1 through 5 with unique clinical descriptions of severity for each adverse event. Protocol-defined dermatologic toxicities include dry skin, pruritus, skin ulceration, acneiform rash, maculopapular rash, skin pain, skin hyperpigmentation, palmar-plantar erythrodysesthesia syndrome, scalp pain, alopecia, paronychia, nail discoloration, nail ridging, and nail loss, which are elaborated in Additional file 1. To reduce bias, the symptoms will be recorded according to therapist diagnosis and patient reports, but will not be graded in the presence of the patients.

##### Skin, nail, and tongue color measurement

In order to evaluate the changes of skin, nail, and tongue color objectively, L*a*b will be used. The L*a*b system, determined by the International Commission on Illumination (CIE) in 1976, is a “gold standard” in assessing color. In the CIELAB color space, values L*, a*, and b* form a three-dimensional coordinate system by plotting at right angles to one another [28]. Value L* represents light/dark and extends from 0 (black) to 100 (white). Value a* represents the red/green axis, and value b* represents the yellow/blue axis. Recently, many methods are available to measure skin color, such as visual assessment, rating scales, photography, reflectance spectroscopy, etc. After analyzing the advantages and disadvantages of the methods, we decided to adopt digital images in this study. Digital image analysis has been used for more than 15 years in medical imaging; it does not directly contact the skin and therefore cannot disturb the measurement of skin color (e.g., by blanching the skin). The digital camera and environment like lighting source and position, background, and distance between camera and subject will be fixed. First, we will use a Canon® digital camera to take pictures. In order to reduce interference from the external environment, a calibration color chart (Spyder CHECKR 24) will be adopted. Then, the Color Sampler tools in Adobe PhotoShop Creative Suite 6 (CS6) will be used for digital image color analysis, which can retrieve and record L*a*b* color values. We will record the skin color of the forehead, right and left cheeks, jaw, and dorsum of the right and left hands, the nail color of ten fingernails, and tongue color.

#### Secondary outcomes

##### Fatigue measurement

Fatigue is an obvious symptom in patients with cancer who are receiving chemotherapy. Fatigue will be measured by the Chinese version of Functional Assessment of Chronic Illness Therapy-Fatigue version 4 (FACIT-F), which can be used with any tumor type [29]. It consists of 40 items scored on a 5-point Likert scale, ranging from 0 to 4. It has five subscales including Physical Well-Being, Social/Family Well-Being, Emotional Well-Being, Functional Well-Being, and Additional Concerns. A total score will be obtained by summing all subscale scores.

##### Clinical objective examination

*Myelosuppression*: There is evidence showing that cancer patients during chemotherapy often have decreased white blood cells (WBCs), hemoglobin (HGB), and platelets (PLTs) [30]. WBCs, also called leukocytes, can be divided into five types, neutrophils, basophils, eosinophils, lymphocytes, and monocytes, which are capable of defending the body against foreign invaders and infectious diseases. The WBC count is often regarded as an indicator of many diseases. During chemotherapy, the decrease of WBC counts in peripheral blood not only lead to fever, but also even septicemia and in severe cases, bleeding, which is one of the reasons for complications after chemotherapy, but these symptoms also disturb the process of chemotherapy, further reducing the efficacy of treatments [31]. HGB is an iron-containing oxygen-transport metalloprotein in the red blood cells of all vertebrates, which is essential for metabolism. Its decrease may result in anemia; the symptoms include shortness of breath, palpitation of the heart, and fatigability [32]. PLTs are derived from megakaryocytes of the bone marrow and only exist in the blood of mammals. The main function of PLTs is to stop bleeding. Chemotherapy-induced significant reduction of PLTs will cause thrombotic thrombocytopenic purpura characterized by the presence of microangiopathic hemolytic anemia, thrombocytopenic purpura, fever, renal abnormalities, and neurological abnormalities [33]. Therefore, the changes of WBC, HGB, and PLT will be assessed.

*Hepatotoxicity*: Studies showed that many patients with cancer experienced liver injury during chemotherapy [34]. From the view of Chinese medicine, the liver and kidneys are the main organs responsible for detoxification. To evaluate liver function objectively, alanine aminotransferase (ALT), aspartate transaminase (AST), total protein, and albumin (Alba) will be measured.

*Nephrotoxicity:* Liver and kidney function are important indicators in evaluating drug safety. Moreover, based on the theory of homogeny of liver and kidney in Chinese medicine, it is necessary to test kidney function. In the testing of kidney function, injury to the kidney can be exactly reflected by creatinine in the blood. The concentration change of creatinine is determined by the glomerular filtration rate, and only at a rate down to one-third of normal will the creatinine increase significantly [35]. In addition, blood urea nitrogen (BUN) is an important indication of renal (kidney) health. Therefore, creatinine (Cr) and BUN will be used as indexes to assess nephrotoxicity.

All participants will be assessed before the intervention (baseline), and 3, 6, and 12 weeks after intervention. All YH0618 materials conform to Food and Environmental Hygiene Department’s requirement for food safety and achieve the certificate of quality. The packing will be conducted at clinics of the School of Chinese Medicine, HKU. A professional research assistant will teach the subjects how to decoct the YH0618 soup, and the specific intake, water amount, and decoction time will be provided to each subject. These subjects will be asked to complete the questionnaires, undergo blood tests by themselves in hospitals, and send all testing results. Quality and compliance to intervention will be achieved by checking attendance records and the diary of self-record kept by each participant. All outcome assessors will be blinded to the intervention types of participants.

### Adverse events

All adverse events should be reported spontaneously by patients or observed by assessors and will be recorded. Adverse events will be graded on a five-point scale (Mild, Moderate, Severe, Life threatening, Causing death). When an adverse event occurs, the investigator will take all necessary and appropriate measures to ensure the safety of the patient. Any questions concerning adverse events associated with the treatment will be reported on the study case report form and sent to the Institutional Review Board of the University of Hong Kong/Hospital Authority Hong Kong West Cluster (HKU/HA HKW IRB).

### Statistical analysis

Statistical analysis will be conducted by a statistician using SPSS version 22.0. Analyses will be performed based on intention-to-treat principles, and any missing data in the follow up visits will be imputed using multiple imputation.

Descriptive statistics will be used to summarize the baseline demographics and clinical characteristics for both groups. Missing values at follow-up visits or drop-outs in the two groups will be assessed to identify any potential bias.

For the primary outcomes, relative risk for the incidence of dermatologic toxicities with grade ≥2, including 95 % CIs obtained via normal approximation to the binomial distribution, will be analyzed for the two groups. The changes in nail color and skin color from baseline to 6 weeks will be compared between the two groups by independent sample *t* tests. Because participants will be randomized into the intervention group or wait-list group, little confounding is expected. However, potential confounding variables will be identified as those that differ among treatment groups at baseline and are significantly associated with outcomes. In case any potential confounders are identified, a logistic regression model and a multiple linear regression model will be used to analyze the binary and continuous outcomes, respectively.

In further analyses using measures at all time points, the relative effectiveness of the interventions, including all primary and secondary outcomes, will be assessed in repeated measures mixed models, using the generalized estimating equation (GEE) modeling method. The repeated measures regression models account for the correlations between individual measurements when repeated over time. The primary test of effect depends upon differing patterns of change (slope) over time; thus, differences in slope will be identified by significant interactions of intervention group with time. If the interaction terms are significant, then further analyses will use contrasts within the models to compare groups at each time point and to compare outcomes across time within each group.

## Discussion

This is the first strict randomized controlled trial to evaluate the effect of medical food on ameliorating chemotherapy-induced toxicity. The proposed study may provide direct and convincing evidence to support YH0618 as an adjuvant treatment for reducing chemotherapy-induced toxicity, which could be introduced into clinical settings. Since little attention has been given to exploring the role of medical food intervention in reducing chemotherapy-induced toxicity, this study will provide solid scientific evidence for international healthcare policy makers to support the integration of alternative intervention strategies in providing care to patients with cancer. When considering that more and more cancer patients are facing chemotherapy-induced side effects even after they have completed chemotherapy, our achievements will provide a safe and effective way for reducing chemotherapy-induced toxicity and improving their quality of life. The objective assessments for chemotherapy-induced toxicity, such as color index, are another important advantage in this study.

However, the main potential limitation of this protocol is that it is not double-blinded. A placebo would not be used in this study, because it is difficult to make a dietary placebo with the limited manpower and finances at this stage. It is also more fair to the wait-list group, as all of the subjects have opportunities to drink the YH0618 soup. If the results are positive, we will further compare the differences of the YH0618 soup and a placebo in order to avoid psychological effects in the future. Although the participants are not blinded, all of the evaluators and statisticians are blinded.

### Trial status

Recruitment started in July 2015, and the trial is expected to be completed in June 2017.

## Abbreviations

Alba, albumin; ALT, alanine aminotransferase; AST, aspartate transaminase; BUN, blood urea nitrogen; CIE, International Commission on Illumination; Cr, creatinine; DSMB, Data and Safety Monitoring Board; FACIT-F, Functional Assessment of Chronic Illness Therapy-Fatigue; GEE, generalized estimating equation; HGB, hemoglobin; HKU/HA HKW IRB, Institutional Review Board of the University of Hong Kong/Hospital Authority Hong Kong West Cluster; NCI CTCAE, National Cancer Institute Common Terminology Criteria for Adverse Events; PLT, platelet; WBC, white blood cell

## Additional file

Additional file 1: Specific criteria for assessing chemotherapy-induced dermatologic toxicity (NCI CTCAE). (DOCX 22 kb)

## References

[1] Steward BW, Wild CP (2014). World cancer report 2014.

[2] Lemieux J, Maunsell E, Provencher L (2008). Chemotherapy-induced alopecia and effects on quality of life among women with breast cancer: a literature review. Psychooncology.

[3] Miller KK, Gorcey L, McLellan BN (2014). Chemotherapy-induced hand-foot syndrome and nail changes: a review of clinical presentation, etiology, pathogenesis, and management. J Am Acad Dermatol.

[4] Lacouture ME, Mitchell EP, Piperdi B, Pillai MV, Shearer H, Iannotti N (2010). Skin toxicity evaluation protocol with panitumumab (STEPP), a phase II, open-label, randomized trial evaluating the impact of a pre-emptive skin treatment regimen on skin toxicities and quality of life in patients with metastatic colorectal cancer. J Clin Oncol.

[5] Grevelman EG, Breed WPM (2005). Prevention of chemotherapy-induced hair loss by scalp cooling. Ann Oncol.

[6] Gilbar P, Hain A, Peereboom VM (2009). Nail toxicity induced by cancer chemotherapy. J Oncol Pharm Pract.

[7] Heidary N, Naik H, Burgin S (2008). Chemotherapeutic agents and the skin: an update. J Am Acad Dermatol.

[8] Crown J, O’Leary M (2000). The taxanes: an update. Lancet.

[9] Crooke ST, Duvernay VH, Galvan L, Prestayko AW (1978). Structure-activity relationships of anthracyclines relative to effects on macromolecular syntheses. Mol Pharmacol.

[10] Pommier Y, Leo E, Zhang HL, Marchand C (2010). DNA topoisomerases and their poisoning by anticancer and antibacterial drugs. Chem Biol.

[11] Pang B, Qiao X, Janssen L, Velds A, Groothuis T, Kerkhoven R (2013). Drug-induced histone eviction from open chromatin contributes to the chemotherapeutic effects of doxorubicin. Nat Commun.

[12] Balagula Y, Rosen ST, Lacouture ME (2011). The emergence of supportive oncodermatology: the study of dermatologic adverse events to cancer therapies. J Am Acad Dermatol.

[13] Kang YK, Lee SS, Yoon DH, Lee SY, Chun YJ, Kim MS (2010). Pyridoxine is not effective to prevent hand-foot syndrome associated with capecitabine therapy: results of a randomized, double-blind, placebo-controlled study. J Clin Oncol.

[14] Lam W, Bussom S, Guan FL, Jiang ZL, Zhang W, Gullen EA (2010). The four-herb Chinese medicine PHY906 reduces chemotherapy-induced gastrointestinal toxicity. Sci Transl Med.

[15] McCune JS, Hatfield AJ, Blackburn AA, Leith PO, Livingston RB, Ellis GK (2004). Potential of chemotherapy–herb interactions in adult cancer patients. Support Care Cancer.

[16] Chan YC, Wu CC, Chan KC, Lin YG, Liao JW, Wang MF (2009). Nanonized black soybean enhances immune response in senescence-accelerated mice. Int J Nanomedicine.

[17] Yim JH, Lee OH, Choi UK, Kim YC (2009). Antinociceptive and anti-inflammatory effects of ethanolic extracts of Glycine max (L.) Merr and Rhynchosia nulubilis seeds. Int J Mol Sci.

[18] Xia Y, Rivero-Huguet ME, Hughes BH, Marshall WD (2008). Isolation of the sweet components from Siraitia grosvenorii. Food Chem.

[19] Liao HF, Chen YJ, Yang YC (2005). A novel polysaccharide of black soybean promotes myelopoiesis and reconstitutes bone marrow after 5-flurouracil-and irradiation-induced myelosuppression. Life Sci.

[20] Wang LS, Stoner GD (2008). Anthocyanins and their role in cancer prevention. Cancer Lett.

[21] Tsoyi K, Park HB, Kim YM, Chung JI, Shin SC, Shim HJ (2008). Protective effect of anthocyanins from black soybean seed coats on UVB-induced apoptotic cell death in vitro and in vivo. J Agric Food Chem.

[22] Choi JH, Choi CY, Lee KJ, Hwang YP, Chung YC, Jeong HG (2009). Hepatoprotective effects of an anthocyanin fraction from purple-fleshed sweet potato against acetaminophen-induced liver damage in mice. J Med Food.

[23] Wang CJ, Wang JM, Lin WL, Chu CY, Chou FP, Tseng TH (2000). Protective effect of Hibiscus anthocyanins against tert-butyl hydroperoxide-induced hepatic toxicity in rats. Food Chem Toxicol.

[24] Ademiluyi AO, Oboh G, Agbebi OJ, Akinyemi AJ (2013). Anthocyanin–rich red dye of Hibiscus sabdariffa calyx modulates cisplatin-induced nephrotoxicity and oxidative stress in rats. Int J Biomed Sci.

[25] Schulz K, Altman D, Moher D, CONSORT Group (2010). CONSORT 2010 Statement: updated guidelines for reporting parallel group randomised trials. BMC Med.

[26] Chan AW, Tetzlaff JM, Altman DG, Laupacis A, Gøtzsche PC, Krleža-Jerić K (2013). SPIRIT 2013 statement: defining standard protocol items for clinical trials. Ann Intern Med.

[27] Park SB, Goldstein D, Lin CSY, Krishnan AV, Friedlander ML, Kiernan MC (2009). Acute abnormalities of sensory nerve function associated with oxaliplatin-induced neurotoxicity. J Clin Oncol.

[28] Weatherall IL, Coombs BD (1992). Skin color measurements in terms of CIELAB color space values. J Invest Dermatol.

[29] Cella DF (1997). Manual of the Functional Assessment of Chronic Illness Therapy (FACIT) scales. Version 4.

[30] Yaal-Hahoshen N, Maimon Y, Siegelmann-Danieli N, Lev-Ari S, Ron IG, Sperber F (2011). A prospective, controlled study of the botanical compound mixture LCS101 for chemotherapy-induced hematological complications in breast cancer. Oncologist.

[31] Chen K, Zhang X, Deng H, Zhu L, Su F, Jia W, Deng X. Clinical predictive models for chemotherapy-induced febrile neutropenia in breast cancer patients: a validation study. Plos One. 2014;9:e96413. 10.1371/journal.pone.0096413 PMC4063732 24945817

[32] Laky B, Janda M, Kondalsamy-Chennakesavan S, Cleghorn G, Obermair A (2010). Pretreatment malnutrition and quality of life — association with prolonged length of hospital stay among patients with gynecological cancer: a cohort study. BMC Cancer.

[33] Liu C, Kallogjeri D, Dynis M, Grossman BJ (2013). Platelet recovery rate during plasma exchange predicts early and late responses in patients with thrombotic thrombocytopenic purpura (CME). Transfusion.

[34] Schaid DJ, Spraggs CF, McDonnell SK, Parham LR, Cox CJ, Ejlertsen B (2014). Prospective validation of HLA-DRB1* 07: 01 allele carriage as a predictive risk factor for lapatinib-induced liver injury. J Clin Oncol.

[35] Inker LA, Schmid CH, Tighiouart H, Eckfeldt JH, Feldman HI, Greene T (2012). Estimating glomerular filtration rate from serum creatinine and cystatin C. N Engl J Med.

