# Supplementary material for: Effect of YH0618 soup on chemotherapy-induced toxicity in patients with cancer who have completed chemotherapy: study protocol for a randomized controlled trial
Source: Trials. 2016 Jul 26;17:354. doi: 10.1186/s13063-016-1443-9 (PMC4960811; doi:10.1186/s13063-016-1443-9)
Supplement: Additional file 1: — Specific criteria for assessing chemotherapy-induced dermatologic toxicity (NCI CTCAE). (DOCX 22 kb) [file 13063_2016_1443_MOESM1_ESM.docx]

| **Grades**  **Additional File 1: Criteria for assessing chemotherapy-induced dermatologic toxicity (NCI CTCAE)** | | | | | | | | | |
| --- | --- | --- | --- | --- | --- | --- | --- | --- | --- |
| **Adverse events** | **1** | **2** | | | **3** | | **4** | | **5** |
| Bullous dermatitis | Asymptomatic; blisters  covering <10% BSA | Blisters covering 10 - 30%  BSA; painful blisters; limiting instrumental ADL | | | Blisters covering >30% BSA; limiting self care ADL | | Blisters covering >30% BSA;  associated with fluid or  electrolyte abnormalities; ICU  care or burn unit indicated | | Death |
| Definition: A disorder characterized by inflammation of the skin characterized by the presence of bullae which are filled with fluid. | | | | | | | | | |
| Dry skin | Covering <10% BSA and no associated erythema or pruritus | Covering 10 - 30% BSA and associated with erythema or pruritus; limiting instrumental ADL | | | Covering >30% BSA and  associated with pruritus;  limiting self care ADL | | **/** | | **/** |
| Definition: A disorder characterized by flaky and dull skin; the pores are generally fine, the texture is a papery thin texture. | | | | | | | | | |
| Pruritus | Mild or localized; topical intervention indicated | Intense or widespread;  intermittent; skin changes  from scratching (e.g., edema, papulation, excoriations, lichenification,oozing/crusts); oral intervention indicated; limiting instrumental ADL | | | Intense or widespread;  constant; limiting self care  ADL or sleep; oral  corticosteroid or  immunosuppressive therapy indicated | | **/** | | **/** |
| Definition: A disorder characterized by an intense itching sensation. | | | | | | | | | |
| Skin ulceration | Combined area of ulcers <1 cm; nonblanchable erythema of intact skin with associated warmth or edema | Combined area of ulcers 1 - 2 cm; partial thickness skin loss involving skin or  subcutaneous fat | | | Combined area of ulcers >2 cm; full-thickness skin loss involving damage to or  necrosis of subcutaneous  tissue that may extend down to fascia | | Any size ulcer with extensive  destruction, tissue necrosis, or  damage to muscle, bone, or  supporting structures with or  without full thickness skin loss | | Death |
| Definition: A disorder characterized by circumscribed, inflammatory and necrotic erosive lesion on the skin. | | | | | | | | | |
| Rash acneiform | Papules and/or pustules  covering <10% BSA, which may or may not be associated with symptoms of pruritus or  tenderness | Papules and/or pustules  covering 10 - 30% BSA, which may or may not be associated with symptoms of pruritus or tenderness; associated with psychosocial impact; limiting instrumental ADL | | | Papules and/or pustules  covering >30% BSA, which may or may not be associated with symptoms of pruritus or tenderness; limiting self care ADL; associated with local  superinfection with oral  antibiotics indicated | | Papules and/or pustules covering any % BSA, which may or may not be associated with symptoms of pruritus or tenderness and are  associated with extensive  superinfection with IV antibiotics indicated; life threatening consequences | | Death |
| Definition: A disorder characterized by an eruption of papules and pustules, typically appearing in face, scalp, upper chest and back. | | | | | | | | | |
| Rash maculo-papular | Macules/papules covering <10% BSA with or without  symptoms (e.g., pruritus,  burning, tightness) | Macules/papules covering 10 - 30% BSA with or without symptoms (e.g., pruritus, burning, tightness); limiting  instrumental ADL | | | Macules/papules covering  >30% BSA with or without  associated symptoms; limiting self care ADL | | / | | / |
| Definition: A disorder characterized by the presence of macules (flat) and papules (elevated). Also known as morbillform rash, it is one of the most common cutaneous adverse events, frequently affecting the upper trunk, spreading centripetally and associated with pruritus. | | | | | | | | | |
| Pain of skin | Mild pain | Moderate pain; limiting  instrumental ADL | | | Severe pain; limiting self care ADL | | **/** | | **/** |
| Definition: A disorder characterized by marked discomfort sensation in the skin. | | | | | | | | | |
| Skin hyperpigmentation | Hyperpigmentation covering <10% BSA; no psychosocial impact | Hyperpigmentation covering >10% BSA; associated psychosocial impact | | | | **/** | | **/** | **/** |
| Definition: A disorder characterized by darkening of the skin due to excessive melanin deposition. | | | | | | | | | |
| Palmar-plantar  erythrodysesthesia syndrome | Minimal skin changes or  dermatitis (e.g,erythema, edema, or hyperkeratosis) without pain | | | Skin changes (e.g., peeling,  blisters, bleeding, edema, or hyperkeratosis) with pain; limiting instrumental ADL | | Severe skin changes (e.g.,  peeling, blisters, bleeding,  edema, or hyperkeratosis)  with pain; limiting self care ADL | | / | / |
| Definition: A disorder characterized by redness, marked discomfort, swelling, and tingling in the palms of the hands or the soles of the feet. | | | | | | | | | |
| Scalp pain | Scalp pain | Moderate pain; limiting  instrumental ADL | | | Severe pain; limiting self care ADL | | | / | / |
| Definition: A disorder characterized by marked discomfort sensation in the skin covering the top and the back of the head. | | | | | | | | | |
| Alopecia | Hair loss of <50% of normal for that individual that is not  obvious from a distance but only on close inspection; a different hair style may be required to cover the hair loss but it does not require a wig or hair piece to camouflage | Hair loss of >=50% normal for that individual that is readily apparent to others; a wig or hair piece is necessary if the patient desires to completely  camouflage the hair loss;  associated with psychosocial impact | | | / | | | / | / |
| Definition: A disorder characterized by a decrease in density of hair compared to normal for a given individual at a given age and body location. | | | | | | | | | |
| Paronychia | Nail fold edema or erythema;  disruption of the cuticle | Localized intervention  indicated; oral intervention  indicated (e.g., antibiotic,  antifungal, antiviral); nail fold edema or erythema with pain; associated with discharge or nail plate separation; limiting  instrumental ADL | | | Surgical intervention or IV  antibiotics indicated; limiting self care ADL | | | **/** | **/** |
| Definition: A disorder characterized by an infectious process involving the soft tissues around the nail. | | | | | | | | | |
| Nail discoloration | Asymptomatic; clinical or diagnostic observations only;  intervention not indicated | | Nail discolorationcovering >50% ; associated psychosocial impact | | **/** | | | **/** | **/** |
| Definition: A disorder characterized by a change in the color of the nail plate. | | | | | | | | | |
| Nail ridging | Asymptomatic; clinical or diagnostic observations only;  intervention not indicated | Distortion of nail shape;associated psychosocial impact | | | **/** | | | **/** | **/** |
| Definition: A disorder characterized by vertical or horizontal ridges on the nails. | | | | | | | | | |
| Nail loss | Asymptomatic separation of the nail bed from the nail plate  or nail loss | Asymptomatic separation of the nail bed from the nail plate or nail loss | | | / | | | / | / |
| Definition: A disorder characterized by loss of all or a portion of the nail. | | | | | | | | | |

**Note:** Grade refers to the severity of the dermatologic toxicity. Any means no symptoms, Mild; asymptomatic or mild symptoms; clinical or diagnostic observations only; intervention not indicated. Grade 2 means Moderate; minimal, local or noninvasive intervention indicated; limiting age-appropriate instrumental ADL (refer to preparing meals, shopping for groceries or clothes, using the telephone, managing money, etc). Grade 3 means severe or medically significant but not immediately life-threatening; hospitalization or prolongation of hospitalization indicated; disabling; limiting self care ADL (refer to bathing, dressing and undressing, feeding self, using the toilet, taking medications, and not bedridden).
